# Advances in the regulatory mechanisms of mTOR in necroptosis

**DOI:** 10.3389/fimmu.2023.1297408

**Published:** 2023-12-18

**Authors:** Yawen Xie, Guoyu Zhao, Xianli Lei, Na Cui, Hao Wang

**Affiliations:** ^1^Department of Critical Care Medicine, Peking Union Medical College Hospital, Chinese Academy of Medical Science and Peking Union Medical College, Beijing, China; ^2^Department of Critical Care Medicine, Beijing Jishuitan Hospital, Capital Medical University, Beijing, China

**Keywords:** mTOR, necroptosis, autophagy, RIPK, ROS

## Abstract

The mammalian target of rapamycin (mTOR), an evolutionarily highly conserved serine/threonine protein kinase, plays a prominent role in controlling gene expression, metabolism, and cell death. Programmed cell death (PCD) is indispensable for maintaining homeostasis by removing senescent, defective, or malignant cells. Necroptosis, a type of PCD, relies on the interplay between receptor-interacting serine-threonine kinases (RIPKs) and the membrane perforation by mixed lineage kinase domain-like protein (MLKL), which is distinguished from apoptosis. With the development of necroptosis-regulating mechanisms, the importance of mTOR in the complex network of intersecting signaling pathways that govern the process has become more evident. mTOR is directly responsible for the regulation of RIPKs. Autophagy is an indirect mechanism by which mTOR regulates the removal and interaction of RIPKs. Another necroptosis trigger is reactive oxygen species (ROS) produced by oxidative stress; mTOR regulates necroptosis by exploiting ROS. Considering the intricacy of the signal network, it is reasonable to assume that mTOR exerts a bifacial effect on necroptosis. However, additional research is necessary to elucidate the underlying mechanisms. In this review, we summarized the mechanisms underlying mTOR activation and necroptosis and highlighted the signaling pathway through which mTOR regulates necroptosis. The development of therapeutic targets for various diseases has been greatly advanced by the expanding knowledge of how mTOR regulates necroptosis.

## Graphical Abstract

Our review summarized the molecular mechanism of necroptosis. mTOR, as a key hub for cell fate, regulates necroptosis by various way. The graphical abstract illustrates the association between mTOR and necroptosis and highlights key signaling events responsible for the transition from apoptosis and autophagy to necroptosis.

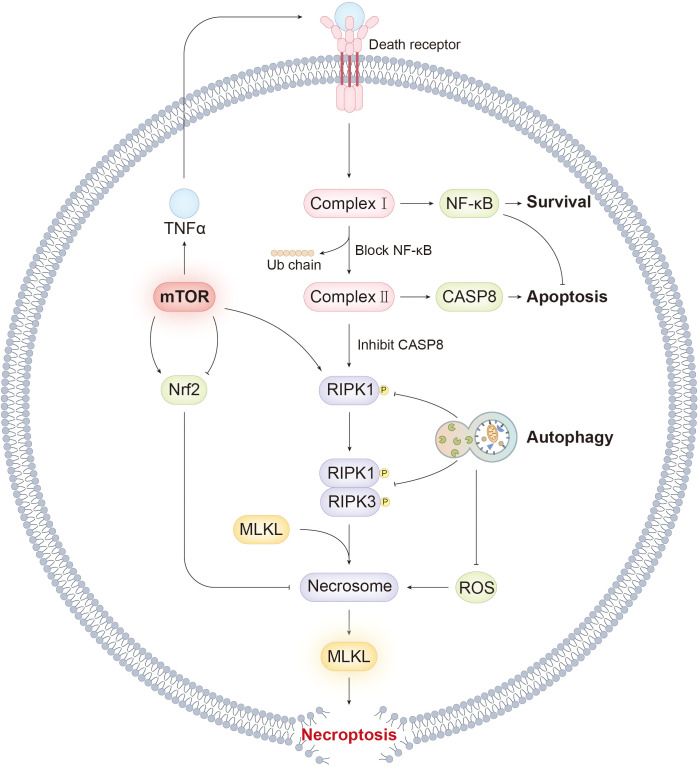

## 1 Introduction

The equilibrium between cell proliferation and death is fundamental for maintaining normal physiological functions. Cell demise facilitates the removal of aging, injured, or dysplastic cells that harm the body’s homeostasis. Hence, vigilant monitoring and elimination of the pernicious cells are essential for development, resistance to various pathogens, and homeostasis maintenance (1). Apoptosis was the first discovered programmed cell death (PCD), whereas necrosis was considered for an extended time to be a typical unprogrammed cell death with no signaling pathway involved. Hirsch et al. found that inhibiting apoptosis may initiate a transition from apoptosis to necrosis, a pivotal milestone in identifying regulated necrotic cell death (2). An increasing number of types of regulated necrosis have been recognized, such as ferroptosis, pyroptosis, and others, among which necroptosis has been investigated most thoroughly (3–5). Necroptosis is characterized by the formation of a necrosome consisting of RIPKs and the activation of MLKL with similar morphological changes to necrosis, including cellular swelling, impairment of plasma membrane integrity, and release of intracellular substances, leading to an inflammatory response (6–8).

Investigating the underlying mechanisms of necroptosis regulation yields potentially effective therapeutic targets. The mammalian target of rapamycin (mTOR), an evolutionally conserved serine/threonine protein kinase, serves as a hub for catabolism and cell growth and plays an essential role in the complex signaling network consisting of numerous regulatory pathways associated with cell death (9). mTOR is involved in the assembly of two different complexes, mTORC1 and mTORC2, by collaborating with other subunits. Given the relatively limited understanding of mTORC2, this review primarily focused on the role of mTORC1. mTORC1 has been identified as an autophagy inhibitor, and increasing evidence indicates that it also regulates necroptosis precisely, thereby identifying new potential therapeutic targets for various disorders (10, 11).

Our objective is to provide a comprehensive literature on the regulatory mechanisms of mTOR in necroptosis by updating a synopsis of recent advances in this area and highlighting clinical applications in necroptosis-related diseases.

## 2 Overview of mTOR

**Figure 1:**
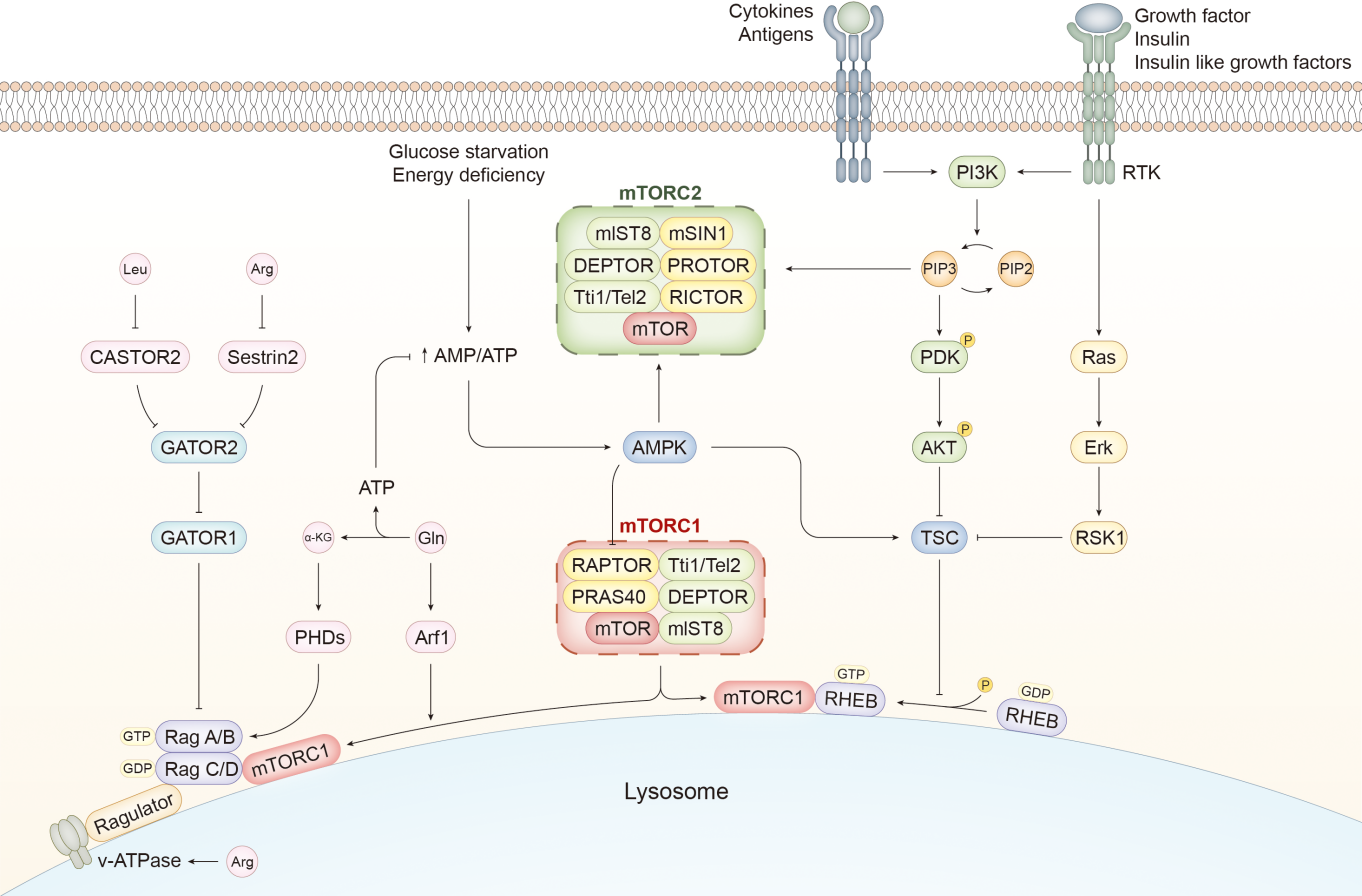
mTORC pathways and activation mechanisms of mTORC1. The components of mTORC1 are slightly different from those of mTORC2. RAPTOR and PRAS40 are unique to mTORC1, whereas mTORC2 contains mSIN1, PROTOR, and RICTOR. Several external stimuli, such as immune signals and growth factors, activate PI3K, which catalyzes the transversion of PIP2 to PIP3. The latter can induce the activation of PDK and AKT by phosphorylation. PIP3 can also activate mTORC2. AKT is a potent stimulator of mTORC1 activity by inhibiting TSC, a GTPase-activating protein. mTORC1 is recruited to the lysosome and activated by RHEB with GTP binding, whereas TSC facilitates the transition from GTP to GDP and suppresses RHEB-mediated mTORC1 activation. In addition to the PI3K/AKT/mTORC1 pathway, growth factors can activate the Ras/Erk/RSK1 pathway to ameliorate the negative effect of TSC on mTORC1 activity. Cellular energetic status is another determiner of mTORC1 activity. AMPK can detect the increasing ratio of AMP to ATP caused by glucose deprivation and inhibit mTORC1 directly and indirectly by phosphorylating RAPTOR and activating TSC, respectively. However, AMPK serves as an activator of mTORC2. In addition to RHEB, mTORC1 is recruited and activated by the Ragulator-Rag complex induced by amino acids. Rag A/B and Rag C/D bound to GTP and GDP, respectively, facilitate the translocation of mTORC1 to the lysosome and activate it with the assistance of Ragulator. CASTOR1 and Sestrin2 sense the alterations in the content of Leucine (Leu) and Arginine (Arg) and mediate the mTORC1-activating signals by suppressing GATOR2, which mitigates the negative effect of GATOR1 on Rag heterodimer. Changes in Arg in the lumen of lysosomes are sensed by v-ATPase, which activates mTORC1 through the Ragulator-Rag pathway. Glutamine (Gln) is converted to α-KG during glutaminolysis, which generates ATP to inhibit AMPK and activate mTORC1. α-KG regulates the nucleotide-binding state of Rag B via PHDs, and Gln enhances the translocation of mTORC1 to the lysosome via Arf1. PI3K, phosphoinositide 3 kinase; RTK, receptor tyrosine kinase; PIP3, phosphatidylinositol 3,4,5 trisphosphate; PDK, phosphoinositide-dependent kinase 1; mTOR, mammalian target of rapamycin; RAPTOR, regulatory-associated proteins of mTOR; PRAS40, 40-kDa pro-rich Akt-substrate; RICTOR, Rapamycin-insensitive companion of mTOR; mSIN1, mammalian stressed-activated map-kinase interacting protein1; PROTOR, protein observed with RICTOR; AMPK, adenosine 5′-monophosphate-activated protein kinase; TSC, tuberous sclerosis complex1; RHEB, Ras homolog enriched in brain; α-KG, α-ketoglutarate; PHD, prolyl hydroxylases; Arf1, ADP-ribosylation factor-1; Leu, leucine; Arg, arginine; Gln, glutamine.

mTOR, an evolutionally conserved kinase, is pivotal in regulating cellular metabolism, growth, and death (9). It was first confirmed 30 years ago by gene screening in budding yeasts and subsequently in mammalian cells (12–16). The mTOR complex and relevant upstream and downstream pathways are discussed in this section (**Figure 1**).

mTOR attaches itself to different components to form mTORC1 and mTORC2, both of which share the same catalytic subunit mTOR and other elements, including mlST8, DEPTOR, and Tti1/Tel2. Regulatory-associated proteins of mTOR (RAPTOR) and the 40-kDa pro-rich Akt-substrate (PRAS40) are the kernels of mTORC1. Rapamycin-insensitive companion of mTOR (RICTOR), mammalian stressed-activated map-kinase interacting protein1 (mSIN1), and protein observed with RICTOR (PROTOR) are exclusive to mTORC2 (17).

Rapamycin, an allosteric inhibitor of mTOR, forms a complex with FK506 binding protein 12 (FKBP12), which can bind to mTOR and hinder the recruitment of the substrate, thereby blocking the interaction between the substrate and the active site of mTOR (18, 19). mTORC1 and mTORC2 have divergent susceptibilities to rapamycin because of the difference between RAPTOR and RICTOR. Rapamycin significantly suppresses mTORC1 but exhibits a relatively mild inhibitory effect on mTORC2, which may be apparent in cell lines or tissues after prolonged treatment (20–22).

### 2.1 Upstream signals and pathways of mTOR activation

Environmental stimuli, immune signals, and metabolic status are the primary upstream signals that activate mTOR (23). Various pathways are associated with mTORC1 activation, such as phosphoinositide 3-kinase (PI3K)/AKT, adenosine 5′-monophosphate-activated protein kinase (AMPK), tuberous sclerosis complex1 (TSC1)/TSC2/Ras homolog enriched in brain (RHEB), VAM6/Rag GTPase, and others (24). RHEB is a GTPase embedded in the lysosome membrane whose activity depends on the type of nucleotide it binds to. mTORC1 can be recruited to the lysosome and activated directly through GTP attachment to RHEB (25, 26).

Environmental stimuli, specifically signals from growth factors, insulin, and insulin-like growth factors, are efficient activators of mTORC1, predominantly mediated by the PI3K/AKT pathway (27). Receptor tyrosine kinases (RTKs) stimulated by growth factors activate PI3K to enable the production of phosphatidylinositol 3,4,5 trisphosphate (PIP3), which transfers the activating signals to phosphoinositide-dependent kinase 1 (PDK1) and AKT (28, 29). TSC acts as a GTPase-activating protein by promoting the conversion of GTP to GDP by irritating the GTPase activity of RHEB to alter its nucleotide binding state to the inactive mode and indirectly inhibits mTORC1 (30). In brief, TSC is suppressed by the PI3K/AKT pathway to facilitate the positive effect of RHEB on mTORC1. Furthermore, downstream of RTKs, the Ras/Erk/p90 RSK1 axis acts as both a direct and indirect activator of mTORC1 by inhibiting TSC and phosphorylating RAPTOR, respectively (31–34).

Antigens, cytokines, and various other immune signals are the main stimulators of mTORC1 activation (35, 36). For example, a toll-like receptor (TLR) is triggered by its legend and conveys the activating signals to mTORC1 through the PI3K/AKT pathway (37, 38).

Considering the critical role of mTOR in metabolism, changes in energy and nutrient abundance are expected to regulate mTORC1. Under the circumstances of glucose starvation or energy depletion, the ratio of AMP to ATP increases due to a decline in ATP, facilitating AMPK activation. AMPK can enhance the inhibitory effect of TSC on mTORC1 by phosphorylating TSC (39). Moreover, it has been shown that mTORC1 is inhibited directly by AMPK by phosphorylating RAPTOR (40).

In addition to glucose, intracellular amino acid levels activate mTORC1, which is mediated by the RAS-related GTP binding protein (RAG)/Ragulator pathway. The heterodimer consisting of RAG A/B and RAG C/D can be ligated to the lysosome with Ragulator as a bridge and transformed to its active state with GTP and GDP attached to RAG A/B and RAG C/D, respectively (41, 42). The RAG heterodimer promotes the transposition of mTORC1 to lysosomes and activates it, whereas GATOR1 suppresses mTORC1 activation by inhibiting RAG A/B (42, 43). GATOR2 can counteract the negative effect of GATOR1 on mTORC1 (43). Sestrin2, when bound to leucine, is deprived of the ability to restrain GATOR2 and mediates the effect of leucine on mTORC1 activation, which gives rise to the interaction between GATOR1 and GATOR2 to activate mTORC1 (44–46). CASTOR1 can detect intracellular arginine, mitigating its suppressive role in GATOR2 to activate mTORC1 indirectly (47). The arginine inside the lysosome activates mTORC1 by interacting with the RAG-Ragulator complex; v-ATPase assists in perceiving changes in arginine levels (48–50). Glutamine activates mTORC1 in both a RAG-dependent and -independent manner. Glutamine is decomposed during glutaminolysis, which yields α-ketoglutarate (α-KG), enabling loading of GTP to RAG B by prolyl hydroxylases (PHDs) (51). The breakdown process also contributes to ATP generation and serves as a bioenergetic resource to ameliorate AMPK-induced mTORC1 inhibition (52, 53). Increasing evidence has substantiated that mTORC1 is activated by glutamine independently of RAG through ADP-ribosylation factor-1 (Arf1), which promotes the recruitment and translocation of mTORC1 to lysosomes (51, 54, 55).

Regarding the mechanisms underlying the activation of mTORC2, it has been well established that PI3K functions upstream of mTORC2 via PIP3, which binds to mSin1 and reverses its inhibitory effect on mTOR to activate it (56). Furthermore, PI3K strengthens the interactions between mTORC2 and the ribosome, which serves as the site of mTORC2 activation (57). In contrast to mTORC1, mTORC2 is activated by AMPK with glucose withdrawal (58). However, more in-depth research on mTORC2 activation is in high demand.

### 2.2 Downstream mechanisms of mTOR

As a key regulator of cellular metabolism and growth, mTORC1 can promote anabolism and inhibit catabolism by controlling the synthesis of proteins, nucleotides, and lipids (59). This part of the review focuses on regulating protein synthesis by mTORC1.

p70-S6 kinase 1 (p70-S6K1) and 4E binding protein 1 (4E-BP1) are the main substrates of mTORC1 and are pivotal to translation. mTORC1 activates p70S6K through phosphorylation, which increases gene expression and protein synthesis by activating various substrates implicated in mRNA maturation and translation. 4E-BP1 inhibits protein production by binding to eukaryotic initiation factor 4E (eIF4E) to block transcription. 4EBP phosphorylated by mTORC1 results in the loss of its affinity for eIF4E, thus reversing its negative effect on transcription and protein synthesis (60–62).

Some substrates of mTORC2 have been identified, including AKT, serum and glucocorticoid-induced protein kinase 1 (SGK1), and protein kinase C-α (PKC-α) (63–65). AKT functions upstream of NF-κB, Bad, and FOXO to regulate cell death. SGK1 and PKC-α activated by mTORC2 are associated with cytoskeleton remodeling and migration (66–68).

## 3 Necroptosis and the crosstalk with other PCDs

### 3.1 Molecular mechanisms of necroptosis

A necrosome composed of RIPKs is required for necroptosis, with activated MLKL serving as the executor of membrane perforation. RIPK1 comprises the N-terminal kinase, intermediate domain, and C-terminal death domain (DD). Several regulatory sites exist in the intermediate domain, such as ubiquitination sites and RIP homotypic interaction motifs (RHIM) (69, 70). RIPK3 and RIPK1 share a similar molecular structure; however, the C-terminal domain of RIPK3 does not encompass DD, which differs from RIPK1 (71). MLKL activated by RIPK3 is integral for the terminal stage of necroptosis. MLKL can form an amyloid-like oligomer that translocates to the plasma membrane and mediates membrane perforation (72, 73).

**Figure 2:**
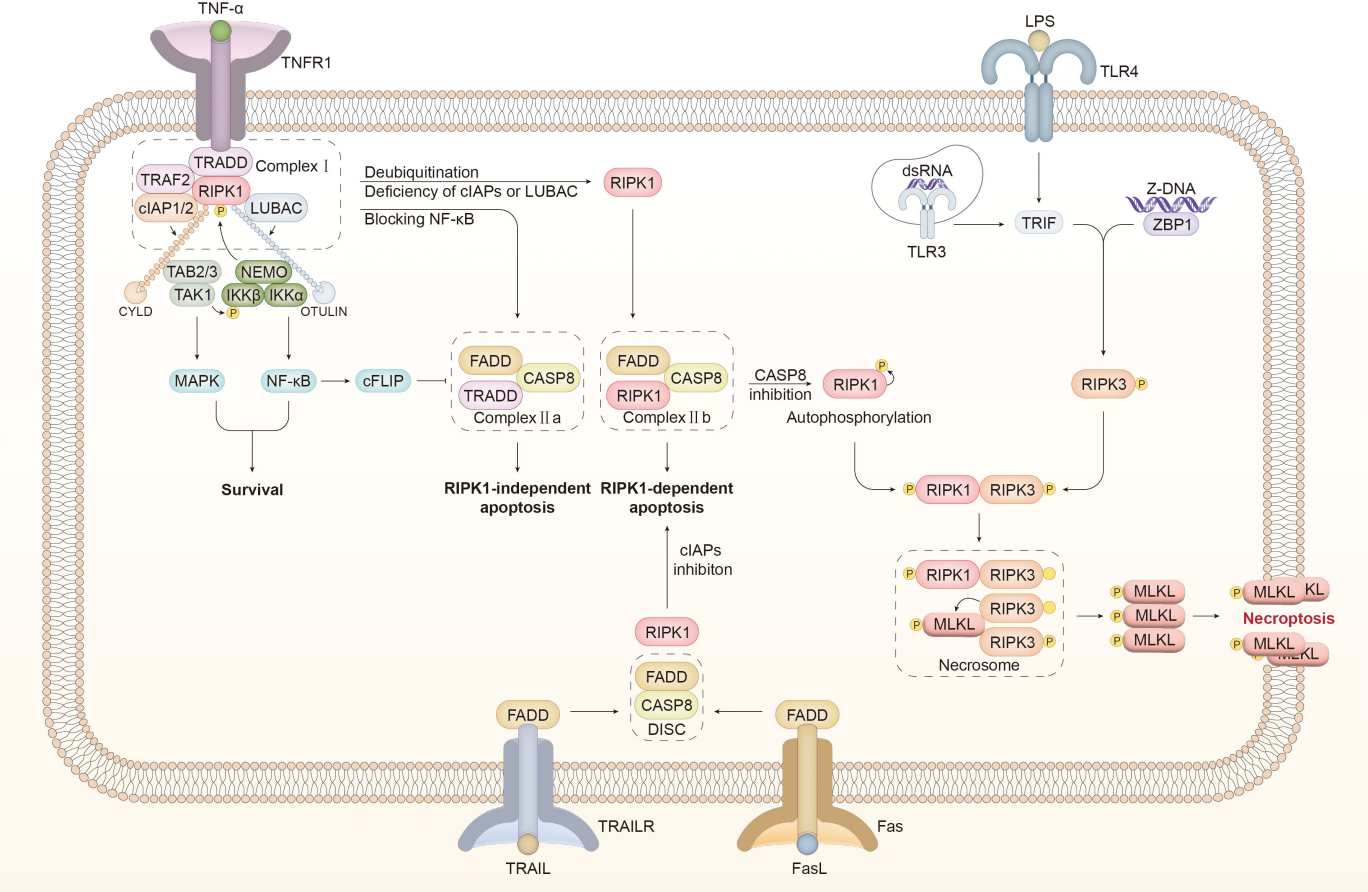
Molecular mechanisms of necroptosis A wide range of signals act as an inducer of necroptosis. TNFR1 bound with TNFα promotes the recruitment of TRADD to assembly complex I, which comprises TRAF2, RIPK1, cIAP1/2, and LUBAC. cIAP1/2 and LUBAC catalyze the connection of K63- and M1-linked ubiquitin chains to RIPK1, which provides a platform for the recruitment of TAK1, TAB2/3, and the IKK complex consisting of NEMO, IKKα, and IKKβ. The kinases mentioned above can inhibit the activity of RIPK1 through phosphorylation to counteract the shift from complex I to complex II. In addition, MAPK and NF-κB signaling pathways are activated downstream of the kinases mentioned above to induce survival. NF-κB facilitates the synthesis of cFLIP, which inhibits the activity of caspase 8 (CASP8). Upon blocking NF-κB, TRADD assembles with FADD and CASP8 to form complex IIa, which mediates RIPK1-independent apoptosis. In certain cases, including cIAP1/2 or LUBAC inhibition and CYLD-mediated RIPK1 deubiquitination, RIPK1 is dissociated from complex I to form complex IIb with FADD and CASP8, inducing RIPK1-dependent apoptosis. Inactivating CASP8 is a prerequisite for the detachment of RIPK1 from complex IIb. RIPK1 is supposed to undergo conformational changes through autophosphorylation and interact with RIPK3 through the RHIM domain. Phosphorylated RIPK3 is oligomerized to recruit and activate MLKL, leading to the assembly of the necrosome. The activated MLKL oligomer is translocated to the cell membrane, mediating membrane perforation and necroptosis. In addition to TNFα, the connection of FasL to Fas and TRAIL to TRAILR transfers a pro-necroptotic signal. DISC comprises FADD and CASP8 to induce apoptosis. Under cIAP1/2 inhibition, DISC associates with RIPK1 to form complex IIb, which can transfer to the necrosome with CASP8 inhibition. TLR3/4 recruits RHIM-containing TRIF in the intracellular domain after dsRNA and LPS are identified, to directly activate the RIPK3/MLKL pathway and thereby trigger necroptosis. ZBP connected to Z-DNA interacts with RIPK3 through the RHIM domain to stimulate downstream MLKL and induce necroptosis. TNF-α, tumor necrosis factor-α; TNFR1, tumor necrosis factor receptor; TRADD, TNF receptor type 1 associated death domain; TRAF2, TNF receptor associated factor 2; RIPK, receptor-interacting serine-threonine kinase; LUBAC, linear ubiquitin chain assembly complex; cIAP1/2, cellular inhibitors of apoptosis 1/2; TAK1, transforming growth factor-β-activated kinase1; TAB2, TAK1-binding protein2; IKKα/β, IKK complex constituted of IκB kinase α/β; NEMO, NF-κB essential modulator; MAPK, mitogen-activated protein kinase; CYLD, Cylindromatosis; OTULIN, OTU deubiquitinase with linear linkage specificity; cFLIP, cellular FLICE-like inhibitory protein; CASP, caspase; FADD, Fas associated death domain; DISC, death-inducing signaling complex; MLKL, mixed lineage kinase domain-like protein; TLR, toll-like receptor; TRIF, Toll/IL-1 receptor domain-containing adaptor protein inducing interferon-β; dsRNA, double-stranded RNA; LPS, lipopolysaccharide; TRAIL, TNF-related apoptosis-inducing ligand.

Necroptosis is initiated by binding the corresponding ligand to the death receptor. Death receptors, belonging to the TNFR (TNF receptor) superfamily, receive and transduce several extracellular signals, including FasL, TNFα, TNF-related apoptosis-inducing ligand (TRAIL), and so on, among which the TNFα-induced mechanism has been studied most thoroughly (74, 75). In addition to death receptors, pattern-recognition receptors (PRRs), such as TLR3 and TLR4, contribute to necroptosis induction by recognizing relevant pathogen-associated molecular patterns (PAMPs) (76, 77). This section provides an overview of the mechanisms of necroptosis induced by various signals (**Figure 2**).

#### 3.1.1 Death receptor-mediated necroptosis

The binding of TNFα to TNFR1 recruits downstream molecules to DD located in the intracellular region of TNFR1, such as TNF receptor type 1 associated death domain (TRADD), TNF receptor-associated factor 2 (TRAF2), and RIPK1. Cellular inhibitors of apoptosis 1/2 (cIAP1/2) are connected to TRADD through TRAF2 (78–80). The binding of TNFR1, TRADD, RIPK1, TRAF2, cIAP1/2, and the linear ubiquitin chain assembly complex (LUBAC) promotes the formation of complex I, resulting in multiple outcomes, including survival, apoptosis, and necroptosis (81, 82).

Complex I-induced cellular survival requires the involvement of ubiquitin ligase. E3 ubiquitin ligases comprising RING E3s, HECT E3s, and RBR E3s are highly substrate-specific. The orientation of the substrate is determined by the conformational flexibility of the substate-binding subunit of E3 ubiquitin ligases (83, 84). cIAP1/2, a homodimeric RING E3 ubiquitin ligase, enables the ligation of the K63-linked ubiquitin chain to RIPK1 on Lys377 (78, 84). LUBAC, another linear ubiquitin E3 ligase, interacts with the K63-linked ubiquitin chain on cIAP1/2 to attach the M1-linked ubiquitin chain to RIPK1 (85, 86). Ubiquitination of RIPK1 catalyzed by cIAP1/2 and LUBAC is propitious for recruiting an array of kinases, including transforming growth factor-β-activated kinase1 (TAK1), TAK1-binding protein2 (TAB2), TAB3, and IKK complex constituted of IκB kinase α (IKKα), IKKβ, and NF-κB essential modulator (NEMO). TAK1 phosphorylates IKKβ to enhance the IKK complex-mediated inhibitory effect on RIPK1 by exerting its kinase activity, which blocks the assembly of complex II and relieves its cytotoxicity. Furthermore, the aforementioned kinases stimulate the NF-κB and mitogen-activated protein kinase (MAPK) signaling pathways, which are both beneficial to cell survival (86–88).

TNFα-induced apoptosis is mediated by complexes IIa and IIb. In addition to activating NF-κB and MAPK pathways, the polyubiquitin chain also interferes with the interplay of RIPK1 and RIPK3 to suppress the transformation from complex I to complex II (89, 90). The deubiquitinating enzyme catalyzes the hydrolysis by which the ubiquitin chain is cleaved from the ubiquitinated protein (91). Cylindromatosis (CYLD), a lysine 63 deubiquitinase, boosts the elimination of the K63-linked ubiquitin chain from RIPK1 and interrupts the NF-κB pathway. OTU deubiquitinase with linear linkage specificity (OTULIN) is another deubiquitinase that exhibits a specific property to degrade the M1-linked ubiquitin chain (92, 93). OTULIN positively affects LUBAC by inhibiting its auto-ubiquitination, and OTULIN deficiency leads to TNFα-induced apoptosis and necroptosis (94, 95). Complex I is destabilized by deubiquitinated RIPK1 or the absence of cIAP1/2, thereby facilitating the transition to complex IIb (96, 97). Cellular FLICE-like inhibitory protein (cFLIP) is structurally analogous to caspase 8 but has no catalytic activity; therefore, it acts as a caspase 8 inhibitor (98). NF-κB activated by complex I can promote the production of cFLIP to inhibit apoptosis (79). Inhibiting the NF-κB pathway or protein synthesis will lead to cFLIP deficiency, which will facilitate the transformation from complex I to complex IIa, induce apoptosis via complete activation of caspase 8, and suppress necroptosis through the cleavage of RIPKs (79, 99). Complex IIa consists of FADD, TRADD, and caspase 8, whereas complex IIb consists of FADD, RIPK1, and caspase 8 (100). When cFLIP production is blocked by NF-κB inhibition, complex IIa leads to RIPK1-independent apoptosis, and RIPK1 phosphorylation on S321 catalyzed by the TAK1 fan facilitates this process. In contrast, RIPK1, with dephosphorylated S321, participates in the assembly of complex IIb (101). DD is essential for RIPK1 dimerization and activation during the transformation process from complex I to complex IIb, contributing to caspase 8 activation by RIPK1 through its kinase domain, thereby inducing RIPK1-dependent apoptosis (101, 102).

The onset of necroptosis is dependent on the formation of complex IIc. Complex II c, also known as necrosome, comprises MLKL, RIPK1, and RIPK3 (100, 103). TNFα combines with TNFR1 to initiate the assembly of complex I. RIPK1 is deubiquitinated by CYLD or is incapable of undergoing ubiquitination with pharmacological or genetic inhibition of cIAP1/2. Both of these conditions are favorable for NF-κB pathway suppression and the transformation from complex I to complex IIa and IIb, which impedes the generation of cFLIP and facilitates apoptosis, respectively. Caspase 8 inactivation or RIPK3 overexpression prevents apoptosis to promote the formation of necrosomes (103). The assembly of necrosomes requires the kinase activity of RIPK1, which is inhibited by necrostatin-1; this inhibitory effect is specific to necroptosis (104, 105). RIPK1 has multiple autophosphorylation sites, among which the N-terminal S161 site is proposed to induce a conformational alteration of RIPK1, leading to the exposure of the RHIM domain and RIPK1 activation (104, 106). Phosphorylated RIPK1 interacts with RIPK3 through the RHIM domain to activate RIPK3 and facilitate the assembly of the RIPK3 oligomer (107). Phosphorylation of Ser 232 of RIPK3 benefits the recruitment of MLKL (108). By phosphorylating MLKL at its Thr357/Ser358 site, RIPK3 facilitates the formation of MLKL oligomer and conformational changes that are essential for its translocation to the membrane and subsequent perforation, which in turn triggers necroptosis (109, 110).

Upon receiving the signal of FasL in conjunction with Fas, FADD is recruited to the intracellular domain of Fas, where it acts as an adaptor for caspase 8 to promote the assembly of death-inducing signaling complex (DISC) and induce extrinsic apoptosis (100). In the absence of cIAPs, RIPK1 is involved in the assembly of complex IIb, which can be converted into a necrosome upon caspase 8 inactivation (100, 111). TRAIL mediates caspase8-dependent apoptosis by recruiting FADD and necroptosis when cIAPs and capsase8 are inhibited, which is similar to FasL (112, 113).

Mounting evidence suggests that reactive oxygen species (ROS) are an important mediator of necroptosis (106, 114). RIPKs are regulated by ROS, which is considered an upstream pro-necroptotic molecule. ROS induces RIPK1 autophosphorylation on S161, which is essential for RIPK1 activation. Autophosphorylated activated RIPK1 is required for RIPK1-RIPK3 co-localization and subsequent necrosome assembly (106). The findings of Bin LU et al. suggested that ROS improves the expression levels of RIPK1 and RIPK3 in glioma cells (115). ROS enhances RIPK1-RIPK3 interaction and necrosome stability, thereby facilitating TNFα-induced necroptosis (115, 116). On the other hand, the production of ROS is also instigated by RIPK3 by activating vital metabolic enzymes (117, 118). Zhentao Yang et al. revealed that RIPK3 boosts aerobic respiration by targeting the pyruvate dehydrogenase complex, which accounts for the increased mitochondrial ROS (mtROS) induction in TNFα-triggered necroptosis (119). The aforementioned findings indicate the existence of a positive feedback regulation loop between RIPKs and ROS during necroptosis. Intriguingly, a recent study by Chi G. Weindel et al. further revealed that mtROS released from gasdermin D (GSDMD)-induced mitochondrial perforation is a potent driver of necroptosis through RIPK3 and that RIPK3 also enhances mtROS production, which indicates that mtROS mediates the pyroptosis-to-necroptosis switch (114).

However, there is a controversy concerning the role of mtROS in necroptosis. Sudan He et al. observed that ROS quenching exerted opposing effects on TNFα-induced necroptosis in L929 and HT-29 cells and concluded that the role of ROS in necroptosis is dependent on cell type (120). Mitochondria depletion blocked necroptosis-related mtROS production but failed to influence TNFα-induced necroptosis dependent on RIPK3, which contradicted the hypothesis that mtROS is a necroptotic trigger (121). Therefore, it is imperative to illustrate the relationship between ROS and RIPKs in necroptosis in various cells and their underlying mechanisms.

#### 3.1.2 PRR-mediated necroptosis

TLR is a main category of PRRs that induce necroptosis when stimulated by pathogen-associated molecular patterns (PAMPs). TLR3 and TLR4 are recognition molecules of viral dsRNA and lipopolysaccharide (LPS), respectively, functioning as a platform to recruit Toll/IL-1 receptor domain-containing adaptor protein inducing interferon-β (TRIF), which contains the RHIM domain, to interact with RIPK3 and induces necrosome-mediated necroptosis (77).

In addition to TLR, Z-DNA binding protein1 (ZBP1) is an RHIM-containing PRR capable of inducing necroptosis. Viral Z-DNA transmits an activation signal to ZBP1, which can directly activate RIPK3 and its downstream effector MLKL in an RIPK1-independent manner. It has been revealed that the mutation in the RHIM domain of RIPK1 results in increased perinatal mortality in RIPK1^RHIM/RHIM^ mice. However, this effect could be reversed by knocking out RIPK3, MLKL, or ZBP1 (122, 123). This finding indicates that RIPK1 inhibits ZBP1-induced necroptosis through the RHIM domain, likely due to the competitive interplay between RIPK1 and ZBP1 with RIPK3.

### 3.2 Conditions for the onset of necroptosis

More novel types of cell death have been discovered gradually, including apoptosis, necroptosis, pyroptosis, ferroptosis, and so on. It is pertinent to emphasize the conditions under which favor necroptosis.

Pan-caspase inhibitor Z-VAD-FMK ignited the switch from apoptosis to necroptosis (2). More initiators of necroptosis have been discovered, which partially overlap with those of extrinsic apoptosis and can be roughly categorized into two groups. One group is dominated by death receptors, including TNFR1, Fas, and TRAILR (72, 124, 125). The other one consists of the receptors which contain the RHIM domain and activate RIPK3 directly (76, 77, 126).

Activated death receptors induce the constitution of complex I (TNFα-TNFR1 and TRAIL-TRAILR) or DISC (FasL-Fas), which triggers extrinsic apoptosis and necroptosis (79). Complex I transfers a pro-survival signal through NF-κB (88). When NF-κB is blocked or RIPK1 is deubiquitinated, complex I shifts to complex II, causing extrinsic apoptosis (127, 128). Inactivating caspase-8 can tip the balance in favor of necroptosis through RIPK1-RIPK3 interaction and necrosome assembly (82, 129). In addition, some RHIM-containing factors activate RIPK3 directly. TLR3 and TLR4 recruit TRIF to activate RIPK3 through the RHIM domain. ZBP1 activated by Z-DNA is another mediator of necroptosis through direct interplay with RIPK3 (130, 131).

### 3.3 Difference of necroptosis and other PCDs

#### 3.3.1 Necroptosis versus apoptosis

Membrane shrinkage and blebbing, chromatin condensation, and DNA fragmentation are typical morphological apoptotic characteristics (132). Apoptotic bodies prevent the inflammatory effect, which is different from necroptosis.

The cascade caspase activation is indispensable for apoptosis. Initiating signals of extrinsic apoptosis are shared with those of necroptosis. The crosstalk between extrinsic apoptosis and necroptosis induced by death receptors has been summarized before. Intrinsic apoptosis is caused by the imbalance of pro-apoptotic BH3-only proteins and anti-apoptotic Bcl-2 proteins, leading to mitochondrial outer membrane permeabilization (MOMP) (133, 134).

The executors of apoptosis and necroptosis are different as well. Necrosome activates and promotes MLKL oligomerization for membrane translocation and perforation (105, 109, 118). Apoptosis is reliant on caspase (135). In intrinsic apoptosis, the substances released from MOMP activate caspase-9 and subsequent caspase-3 and -7 (136). Caspase-8 activated in extrinsic apoptosis not only contributes to the caspase-3 and -7 but also triggers MOMP by cleaving BH3-only proteins (137). Caspase-3 and -7 activate the downstream DNase or undermine the electron transport chain to implement apoptosis (138, 139).

#### 3.3.2 Necroptosis versus pyroptosis

Similar to necroptosis, pyroptosis is another necrotic PCD featured by Gasdermin-mediated membrane perforation and release of proinflammatory cytokines. The inflammasome is the key for pyroptosis, rather than necrosome in necroptosis.

Pyroptosis is reliant on caspases to facilitate the cleavage of pyroptotic executors, which is different from necroptosis. PRRs activate NF-κB to promote the transcription of pyroptosis-related molecules, including NOD-, LRR- and pyrin domain-containing protein 3 (NLRP3), pro-IL-18, and pro-IL-1β (127, 140). NLRP3 activating signals include K^+^ efflux, Ca^2+^ mobilization, lysosomal enzymes, mitochondrial DNA, and ROS (141–145). NLRP3 inflammasome cleaves GSDMD, pro-IL-18, and pro-IL-1β through caspase-1 to induce pyroptosis in the canonical inflammasome pathway (146). Caspase-4/5/11 is activated by LPS to cleave GSDMD and mediate non-canonical pyroptosis (147–149).

The cleavage product, GSDMD-N, is the pyroptosis executor by perforating membrane (150–152). Pro-IL-18 and pro-IL-1β are cleaved and released through the membrane pore (153). Despite that necroptosis and pyroptosis are both characterized by membrane perforation, the mechanisms are distinct and mediated by MLKL and GSDMD respectively.

#### 3.3.3 Necroptosis versus ferroptosis

Ferroptosis is a type of programmed necrosis driven by lipid peroxidation characterized by the involvement of iron overload. The imbalance of oxidation and anti-oxidation systems is the foundation of ferroptosis.

Firstly, the engagement of iron is an apparent difference between ferroptosis and necroptosis. Fe^3+^ combined with the transferrin is conjugated to the transferrin receptor and internalized to be reduced to Fe^2+^ (154, 155). Fe^2+^ can replenish the labile iron pool to participate in lipid peroxidation by iron-dependent Fenton reaction or bind to ferritin to prevent oxidative cytotoxicity (156). Secondly, the antioxidant system is another integral part of ferroptosis but seems to be less essential in necroptosis. System Xc-glutathione (GSH)-GPX4 axis is the predominant antioxidant system for inhibiting ferroptosis (157, 158). What’s more, it is suspected that lipid peroxidation leads to the ultimate cell death but the inner mechanisms have not been elucidated yet (4, 156). Compared to ferroptosis, necroptosis-mediated cellular damage is more highly MLKL-dependent and less reliant on lipid peroxidation.

## 4 Mechanisms of mTOR regulating necroptosis

### 4.1 Autophagy

**Figure 3:**
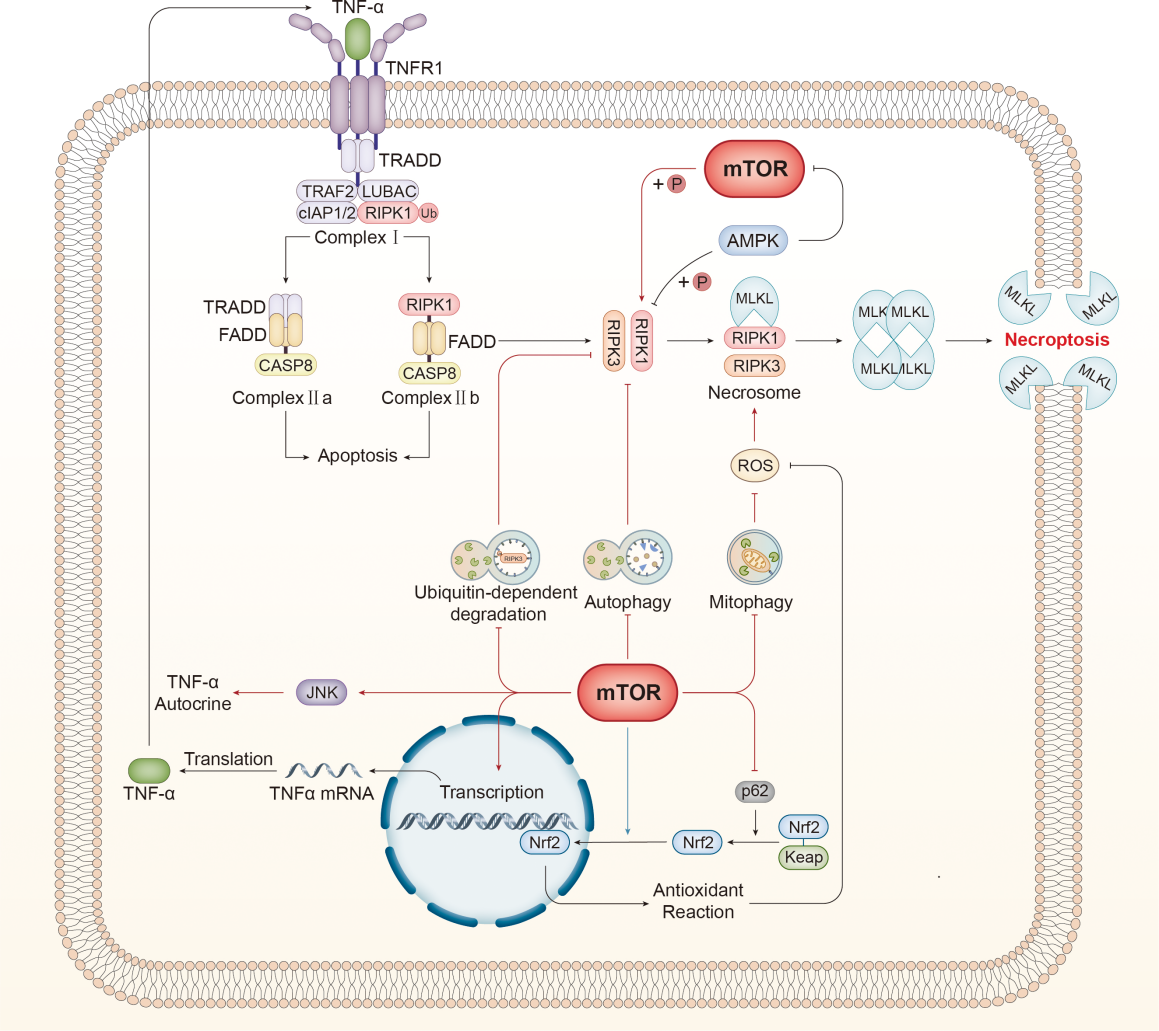
mTOR regulates necroptosis via various mechanisms. mTOR inhibits autophagy, which suppresses necroptosis by disturbing the interaction between RIPK1 and RIPK3. Autophagy specifically recognizes and degrades ubiquitinated RIPK3 and negatively affects necroptosis. Furthermore, damaged mitochondria are the primary source of ROS, which can activate RIPK1 directly and promote the interaction between RIPK1 and RIPK3, facilitating the assembly of necrosomes. Mitophagy restrains ROS production by eliminating injured mitochondria. Furthermore, RIPK1 can be activated by mTOR directly through phosphorylation. mTOR can elevate the level of TNRα mRNA and promote the autocrine effect of TNFα by activating the JNK pathway, both of which can boost necroptosis. Tipping the balance of the redox reaction is another approach for mTOR to regulate necroptosis. p62 assists with the release and stabilization of Nrf2 by counteracting Keap1, and the released Nrf2 translocates to the nucleus and governs the expression of genes associated with antioxidant defense to antagonize necroptosis. mTOR displays bidirectional effects on this process. On the one hand, mTOR inhibits p62 to promote necroptosis, thereby suppressing the antioxidant reaction. In contrast, mTOR promotes nuclear translocation to inhibit necroptosis. TNF-α, tumor necrosis factor-α; TNFR1, tumor necrosis factor receptor; TRADD, TNF receptor type 1 associated death domain; TRAF2, TNF receptor associated factor 2; RIPK, receptor-interacting serine-threonine kinase; LUBAC, linear ubiquitin chain assembly complex; cIAP1/2, cellular inhibitors of apoptosis 1/2; TAK1, transforming growth factor-β-activated kinase1; TAB2, TAK1-binding protein2; IKKα/β, IKK complex constituted of IκB kinase α/β; NEMO, NF-κB essential modulator; MAPK, mitogen-activated protein kinase; CYLD, Cylindromatosis; OTULIN, OTU deubiquitinase with linear linkage specificity; cFLIP, cellular FLICE-like inhibitory protein; CASP, caspase; FADD, Fas associated death domain; DISC, death-inducing signaling complex; MLKL, mixed lineage kinase domain-like protein; TLR, toll-like receptor; TRIF, Toll/IL-1 receptor domain-containing adaptor protein inducing interferon-β; dsRNA, double-stranded RNA; LPS, lipopolysaccharide; ROS, reactive oxygen species; Nrf2, nuclear factor-erythroid 2-related factor 2; Keap1, Kelch-like ECH-associated protein 1.

Autophagy plays an integral role in the regulation of necroptosis. mTORC1 has been universally recognized as an autophagy inhibitor through various mechanisms, such as phosphorylating unc-51-like kinase 1 (ULK1), interrupting transcription factor EB (TFEB) translocation to nuclear, and interfering with lysosome reformation (159, 160). Thus, autophagy serves as a bridge for mTOR to regulate necroptosis (**Figure 3**).

Autophagy disrupts the interaction between RIPK1 and RIPK3 to inhibit necroptosis. Di Ge et al. verified that 11-methoxytabersonine (11-MT) induced necroptosis in A549 and H157 cell lines, and autophagy was also initiated by activating the AMPK/mTORC1 pathway (161). 3-methyladenine (3-MA) and chloroquine (CQ), both of which inhibit autophagy, were utilized to investigate the relationship between autophagy and necroptosis in detail. The combined administration of 11-MT and autophagy inhibitors may intensify the interaction between RIPK1 and RIPK, thereby accelerating necroptosis, which confirmed that mTORC1 promoted necroptosis by downregulating autophagy (161).

Autophagy also has a prominent influence on the levels of cellular RIPKs to inhibit necroptosis. RIPK1 and MLKL are suppressed by increased autophagy resulting from inactivated PI3K/AKT/mTOR/p70S6K pathway (162). AMPK-mTOR is a potential target for treating spinal cord injury (SCI) (163). Inactive mTOR dephosphorylates TFEB to promote the expression of autophagy-related genes and elevated autophagic flux results in the decline of RIPKs and MLKL, which inhibits necroptosis and facilitates the neurofunction restoration (163). However, another study about SCI yielded the opposite conclusion that autophagy may deteriorate the necroptosis-mediated injury because rapamycin antagonizes the edaravone-induced downregulation of phosphorylated necroptotic proteins through autophagy (164). The discrepancy probably derives from different cell types because neurons are the focus in the former, while human brain microvascular endothelial cells are targeted in the latter. Therefore, the effect of autophagy on necroptosis seems to be context-dependent and cell-type specific.

mTORC1 regulates ubiquitin-dependent RIPK3 degradation through autophagy. Yadong Xie and his colleagues found that mTOR hyperactivated by the Western diet or TSC1 knockout caused substantial RIPK3 accumulation through the blockade of autophagy in intestinal epithelial cells, and p62 was involved in the autophagic degradation of RIPK3 as a specific recognizer for ubiquitinated cargo (11). TRIM11, an E3 ubiquitin ligase, catalyzes polyubiquitination of RIPK3 for autophagic degradation, which was abrogated by mTORC1 overexpression (11). Consequently, this study revealed two mechanisms by which mTOR upregulates necroptosis in an autophagy-dependent manner. Kevin Bray et al. (165) also verified that mTOR inhibits the autophagic degradation of ubiquitinated RIPK3 and interferes with TRIM11 to hinder RIPK3 ubiquitination modification to protect RIPK3 from p62-mediated autophagic degradation.

Mitophagy, another vital subtype of autophagy, has been demonstrated to be involved in necroptosis. Especially the damaged mitochondria are the main site of ROS production, which is closely associated with necroptosis. Mitophagy can eliminate injured or fragmentized mitochondria to restrain the production of ROS, thereby preventing necroptosis. Bo Xu et al. revealed that exosomes produced from Schwann cells induced AMPK-mediated mitophagy to subdue the generation of ROS and alleviate necroptosis in PC12 cells but failed to explore the role of mTORC1 in the mechanisms related to mitophagy and necroptosis despite the close relationship between AMPK and mTORC1 (166). Mitochondrial permeability transition pore opening, ROS production, and mitochondrial fission are considered mediators of necroptosis evidence by phosphorylated MLKL locating mitochondrial fractions, which can be alleviated by rapamycin through mitophagy-dependent removal of impaired mitochondria (167, 168). A recent study found that a triggering receptor expressed on myeloid cells-1 (TREM-1) induced necroptosis in macrophages by activating mTOR. The study further concluded that mTOR could elevate the expression levels of mitochondrial fission process protein 1 (MTFP) and phosphatase phosphoglycerate mutase family member 5 (PGAM5) to activate dynamin-related protein 1 (DRP1)-mediated mitochondria fission and subsequent mitophagy (169). Despite the limited evidence for this recently identified pathway, it remains a plausible mechanism by which mTOR regulates necroptosis in a mitophagy-dependent manner.

Nevertheless, there is more than one signaling pathway that connects mitophagy and necroptosis, and mitophagy may potentially serve a pro-necroptotic effect via certain mechanisms rather than an anti-necroptotic role. For instance, Sorafenib has been found to induce mitophagy through the mTORC1-TFEB pathway, and mitophagy-mediated MFN degradation contributed to the increased expression of MAM constituents and induced excessive contact between mitochondria and the ER, an intracellular calcium pool. The process mentioned above resulted in Ca^2+^ flow from the ER to the mitochondria, and this Ca^2+^ overload triggered necroptosis (170).

### 4.2 Expression and phosphorylation modification of key molecules

The key molecules that play a crucial role in necroptosis are directly regulated by mTOR (**Figure 3**). The kinase activity of mTORC1 is effectively utilized to phosphorylate RIPK1, activating RIPK1 and subsequent regulation of necroptosis. Abe et al. observed that rapamycin, an mTOR inhibitor, exhibited a protective effect on cardiomyocytes against necroptosis induced by TNFα/z-VAD-fmk (zVAD) (171). Subsequent investigations have indicated that mTORC1 selectively phosphorylates different RIPK1 sites (e.g., Ser166 and Ser320) and that phosphorylation of these two sites is positively and negatively correlated with RIPK1 activity, respectively (171). Rapamycin promoted the phosphorylation at Ser320 and decreased phosphorylation at Ser166, thereby impairing the activity of RIPK1. Given that Ser320 is required for the reciprocal interaction between RIPK1 and RIPK3, phosphorylation of Ser320 induced by mTOR inhibitor hindered the RIPK1-RIPK3 connection (171). Consequently, mTORC1 facilitates necroptosis by regulating RIPK1 activity and the interaction between RIPK1 and RIPK3 through phosphorylation.

As AMPK has a well-established role as a mTORC1 inhibitor, RIPK1 phosphorylation by AMPK has been increasingly recognized. Tao Zhang et al. uncovered that AMPK stimulated by glucose deprivation restricts RIPK1 activation by phosphorylating RIPK1 S415, thereby inhibiting necroptosis during short-term energy stress (172). Prolonged 2-DG treatment to mimic long-term glucose starvation leads to a decrease and an increase in p-RIPK1 at Ser^415^ and Ser^166^, respectively, which implies that AMPK mediates the conversion from cellular protection to necroptosis by regulating RIPK1 phosphorylation at different sites in the context of glucose deficiency (172). However, another study reported that activated AMPK inhibits necroptosis through the enhancement of pro-caspase 8 stability, rather than regulating the level of p-RIPK1 in acute pancreatitis mice models (173). The divergent conclusions mentioned above can be attributed to the different activators of AMPK and the cell types. Different metabolic status leads to diverse types of cell death in different cells. Consequently, it is necessary and meaningful to investigate the relationship between metabolic status and necroptosis and the connections between AMPK and RIPK1 in necroptosis.

It is widely recognized that TNFα is a crucial signal that initiates necroptosis. Consequently, mTOR also targets necroptosis to modulate it. It was demonstrated that TNFα/z-VAD-fmk (zVAD)-induced necroptosis was significantly suppressed by a PI3K/mTOR inhibitor or miRNA-mediated mTOR gene silencing (174). An increase in TNFα mRNA was detected in the cells undergoing necroptosis, suggesting that necroptosis was accompanied by a significant rise in TNFα synthesis and secretion. To elucidate the inherent relationship between mTOR and TNFα, researchers used a combination of PI3K/mTORC1 inhibitor and miRNA knockdown of mTOR or AKT. Their findings revealed that TNFα mRNA was significantly decreased in the absence of mTOR, which indicated that PI3K/AKT/mTORC1 could upregulate the synthesis of TNFα (174). c-Jun N-terminal kinase (JNK) is involved in TNFα synthesis and autocrine and is a downstream effector of mTOR to regulate necroptosis (175–177). McNamara et al. found that the level of active JNK relevant to necroptosis increased in cells where AKT/mTOR was suppressed by siRNA knockdown or inhibitors. This finding suggested that TNFα autocrine was promoted through AKT/mTOR/JNK to exacerbate the necroptosis and establish a positive feedback loop (174). In addition to TNFα, it is noteworthy that TRAIL, another necroptosis inducer, may be associated with TSC2 and mTOR. A recent study revealed that TSC2 facilitates the mTORC2-skewed profile in malignant cells to defend against the attack from cytotoxic T cells. TSC2 ablation leads to mTORC1 overactivation and increased expression of TRAIL receptor to increase the susceptibility to necroptosis, indicating the close relationship between TSC2-mTOR and TRAIL signaling and their pivotal role in necroptosis (178).

Moreover, it’s conceivable that mTORC1 is also regulated by necroptosis-related molecules. Rune Busk Damgaard et al. used hepatocyte-specific knockout of OTULIN (*Otulin*^Δhep^) mice to establish that the lack of OTULIN led to mTORC1-associated steatohepatitis, hepatic fibrosis, and cancer (179). Abnormal mTORC1 activation was indicated in *Otulin*^Δhep^ mice, and the administration of the mTORC1 inhibitor Rapamycin alleviated the liver injury and pathology caused by OTULIN insufficiency, which might be associated with TSC and RHEB. However, the intrinsic mechanism by which OTULIN regulates mTORC1 and its contribution to necroptosis still requires further research.

### 4.3 Oxidative stress

ROS is regarded as a signal of necroptosis (180–182). Mechanically, RIPK1 autophosphorylation and activation are promoted by ROS, which facilitates the recruitment of RIPK3 and the assembly of necrosomes. Thus, oxidative stress connects mTOR to necroptosis through ROS (**Figure 3**).

mTOR regulates the generation of ROS. Q Liu et al. revealed that the pharmacological or genetic blockade of AKT/mTOR undermined the production of ROS without affecting the interaction between RIPK1 and RIPK3 in HT22 cells treated with TNFα/zVAD to alleviate necroptosis, indicating that AKT/mTOR functioned downstream of RIPK1 but upstream of ROS generation (183). Forkhead box subclass O (FOXO) transcription factors, which govern antioxidant defenses, were substantiated to be involved in the regulatory mechanism of AKT/mTOR regarding redox reaction and oxidative stress (183, 184). FOXO phosphorylated by AKT is deprived of pro-transcription capacity and is removed from the nucleus when the 14-3-3 protein binds to it (185). Sustained AKT/mTOR inhibition relieved the phosphorylation suppression of FOXO and facilitated antioxidant response to downregulate necroptosis (183). Since AKT has been widely acknowledged as an upstream inhibitor of FOXO, mTOR, a downstream molecule of AKT, still warrants further exploration for its relationship with FOXO and potential regulatory mechanism.

Nuclear factor-erythroid 2-related factor 2 (Nrf2) is another target of mTOR to regulate oxidative stress. Similar to FOXO, Nrf2 is another transcription factor that controls the expression of antioxidant genes. Kelch-like ECH-associated protein 1 (Keap1), a ubiquitin ligase complex, binds to Nrf2 to facilitate its degradation by ubiquitination. p62 has an affinity for Keap1 and is therefore considered a guardian of Nrf2. Keap1 is inhibited upon binding to p62, facilitating the release and stabilization of Nrf2 for translocation to the nucleus and subsequent antioxidant response (186, 187). It has been revealed that the mTOR inhibitor CCI-779 facilitated the dissociation of Nrf2 from Keap1 by upregulating p62 to enhance the antioxidant reaction but blocked the nuclear translocation of Nrf2 to prohibit antioxidant defense (165).

Intriguingly, the pro-necroptotic and anti-necroptotic roles of mTOR were well illustrated in this study (165). In contrast to the views of the majority of the studies above, this study concluded that the mTOR inhibitor-induced necroptosis through different mechanisms. However, it is not contradictory because the regulatory network between mTOR and necroptosis is complicated by the intersection of numerous signaling pathways. The positive or negative regulatory effect of mTOR on necroptosis is determined by the predominant mechanism, which may be influenced by various factors, including cell type and stimulus. Kevin Bray et al. demonstrated that the effect of mTOR inhibitors blocking the nuclear translocation of Nrf2 was more significant than inducing mitophagy to eliminate ROS and relieving Nrf2 from Keap1, which was ultimately manifested as the promotional role of mTOR inhibitors on necroptosis (165).

## 5 Advances in the therapeutic potential of mTOR in preclinical research

### 5.1 Immune-related disorders and infectious diseases

Necroptosis considered a proinflammatory cell death, has been authenticated to be associated with a variety of diseases. We will discuss potential implications in immune-related disorders and infectious diseases by mTOR through necroptosis regulation, such as autoimmune disease, sepsis and related complications, graft rejection, and infection (131, 188–190).

The AKT/mTOR pathway promotes TNFα-induced necroptosis by increasing TNFα production and activating JNK, both of which facilitate TNFα autocrine and deteriorate the damage caused by necroptosis (174). The finding that mTOR regulates necroptosis through TNFα implies that the blockade of the signaling pathway could be a potential direction in suppressing pathologic inflammation and consequently alleviate the inflammatory injury, which is particularly beneficial in treating acute pancreatitis, bacterial infections and systemic inflammatory response syndrome (SIRS). Zhong et al. identified a novel mechanism by which mTORC1 promoted DRP1-mediated mitochondrial fission through downstream molecules, PGAM5 and MTFP, thereby aggravating the inflammatory damage induced by necroptosis in alveolar macrophages treated with LPS, indicating a new therapeutic target for LPS-triggered acute lung injury (169).

The pathogenesis of IBDs, including Crohn’s disease and ulcerative colitis, is related to the necroptosis of intestinal epithelial cells, a process in which mTOR has been identified as a participant. Xie et al. revealed that mTOR overactivation promoted necroptosis of epithelial cells to damage the intestinal barrier and induce intestinal inflammation and inflammation-related cancer (11). Mechanically, mTOR inhibited autophagic degradation of RIPK3 by downregulating RIPK3 ubiquitination to promote necroptosis, which provided more precise therapeutic directions for IBDs (11). Moreover, the role of necroptosis in other autoimmune diseases has been validated, including autoimmune hepatitis, lupus nephritis, and autoimmune lymphoproliferative syndrome (191–194). However, investigations into the potential therapeutic value of mTOR in the aforementioned autoimmune disorders through necroptosis are lacking.

It has been demonstrated that mTOR plays a role in immune defense against pathogens by regulating necroptosis, which provides a possible target for treating infectious diseases. Macrophages infected with Candida albicans, a common fungal pathogen, undergo MLKL-mediated necroptosis considerably. mTORC1 contributes to the activation of RIPK1, RIPK3, and MLKL and specific knockout of Tsc1 in macrophage/neutrophil gives rise to the prominent increase in fungal burden and cell death, suggesting the potential of TSC1-mTORC1 in defending pathogen infections (195). Moreover, AKT/mTOR is inhibited by ursolic acid to promote autophagy and suppress the necroptosis of macrophages, enhancing the anti-infection effect against Mycobacterium tuberculosis (196). Nevertheless, further studies are required to validate the role of mTOR-regulated necroptosis in other pathogen infections, such as SARS-CoV-2.

In terms of graft rejection, substantial evidence proves the function of necroptosis in organ transplantation (190, 197–199). However, only one study observed that the cardiac allograft models survived longer with the treatment of mTOR inhibitor sirolimus, which may be associated with necroptosis (190). Therefore, more research is in demand to explore whether and how mTOR is engaged in regulating necroptosis in graft rejection.

### 5.2 Tumor

The regulatory mechanism of mTOR on necroptosis benefits the treatment of various tumors. It has been verified that auranofin can induce necroptosis by inhibiting the PI3K/AKT/mTOR pathway to restrain the exacerbation of non-small cell lung cancer (NSCLC); however, additional research is needed to elucidate the underlying mechanism (200). In lung cancer, the combination administration of 11-MT and an autophagy inhibitor exhibited more efficient therapeutic benefits compared to 11-MT used alone. This can be attributed to the finding that the AMPK/mTOR pathway inhibited the interaction between RIPK1 and RIPK3 via autophagy, suppressing necroptosis (161). Moreover, the therapeutic value of regulating necroptosis via mTOR has also been confirmed in schwannoma. Necroptosis of schwannoma cells is induced by LiCl through the AKT-mTOR-p70S6K axis to restrain the deterioration of schwannoma (201).

Regarding urinary tumors, the role of mTOR in regulating necroptosis has been gradually realized. Jin et al. used GNE-493 to reveal a new therapeutic target for prostate cancer by blocking the PI3K-AKT-mTOR pathway and inducing ROS production which brought about oxidative damage and necroptosis in prostate cancer cells (202). In renal cell carcinoma, an mTOR inhibitor has been demonstrated to promote autophagy-mediated RIPK clearance to inhibit necroptosis, but it also exerts a positive role in necroptosis by suppressing Nrf2 nuclear translocation and consequent antioxidant defense. Therefore, autophagy inhibitors are expected to enhance the efficacy of mTOR inhibitors in treating renal cell cancer (165).

### 5.3 Other diseases relevant to necroptosis

There are numerous poisonous agents. For instance, Sorafenib is a targeted drug for hepatocellular carcinoma but is restricted in its clinical applicability due to its myocardial toxicity to some extent (203, 204). mTOR can be targeted to protect the myocardium from Sorafenib, which was validated to degrade MFN2 through mTOR-regulated mitophagy, disrupting the balance of mitochondrial Ca^2+^ and inducing necroptosis (170). Therefore, mTOR represents a potentially effective treatment for myocardial damage induced by Sorafenib. Additionally, saturated fatty acids can trigger necroptosis in cardiomyocytes to provoke oxidative damage (205). Mingyue Zhao et al. found that the AKT/mTOR signaling pathway activated by palmitic acid elevated the levels of RIPK1 and RIPK to induce the necroptosis of cardiomyocytes, which is likely to induce myocardial hypertrophy. They concluded that mTOR inhibition could reverse necroptosis triggered by increased saturated fatty acid to treat myocardial hypertrophy (206).

Despite most studies holding the view that mTOR is indispensable in promoting necroptosis, a few studies have reached the opposite conclusion. Intake of the plastic degradation product microplastic (MP) and plastic additive Di (2-ethylhexyl) phthalate (DEHP) can elicit the toxicity of skeletal muscle. Liu et al. investigated its potential mechanisms and proposed that DEHP/MP triggered necroptosis by inhibiting the PI3K/AKT/mTOR signaling pathway and stimulating oxidative stress, which suggested that mTOR could be targeted to curtail the plastic-induced muscle toxicity (207).

An increasing number of studies have substantiated the role of necroptosis in neurodegenerative diseases, such as Alzheimer’s disease (AD), Parkinson’s disease (PD), Multiple Sclerosis, and amyotrophic lateral sclerosis (ALS) (208–211). However, explorations into the therapeutic utility of mTOR-regulated necroptosis in these disorders are lacking. Indirect evidence indicates the treatment potential to some extent. p62 is involved in the TNFα-induced necroptosis by interacting with RIPK1 in AD, which is aggravated by comprised autophagy through aberrant p62 accumulation (212). As a well-established autophagy inhibitor, mTOR is likely to regulate necroptosis through the autophagy-p62-RIPK1 axis and serve as a potential therapeutic target in AD. A recent study found that the activation of necroptosis by the long noncoding RNA *MEG3* is associated with AD (213). The strong correlation between *MEG3* and mTOR activity has been established in numerous diseases, including neuroblastoma, endometrial carcinoma, and cisplatin-induced nephrotoxicity (214–217). Thus, mTOR may mediate *MEG3*-induced necroptosis. Due to the lack of direct evidence, further investigations are urgently required.

In other neurological diseases, a study revealed that AKT/mTOR contributed to ROS production to exacerbate necroptosis in the mouse hippocampal neuronal cell line HT-22. However, the study did not delve deeper into the potential involvement of this mechanism in neurological diseases through *in vivo* experiments (183). Ying Wang et al. revealed the treatment mechanisms of LiCl in schwannoma by activating AKT/mTOR axis to induce necroptosis (201).

## 6 Therapeutic implications of mTOR inhibitors in clinical trials

Since the therapeutic potentials reviewed above are shed light on by animal models or *in vitro* experiments, it is obligatory to keep track of the advances in the clinical trials of mTOR inhibitors with verified necroptosis-regulating roles.

A wide range of basic research has confirmed that various mTOR inhibitors have the potential to regulate necroptosis, which has been summarized before in this review. Rapamycin (also named sirolimus) has been substantiated to regulate necroptosis as a well-characterized mTOR inhibitor. For instance, the treatment of rapamycin mitigates the injury of intestinal epithelial cells by recovering the mitophagy flux to alleviate necroptosis, which is verified in neurons, macrophages, cardiomyocytes, and so on (167, 169, 218, 219). Additionally, other mTOR inhibitors exert important effects on necroptosis, such as AZD2014 (also named vistusertib), everolimus, and Torin1 (171, 220). The advances in clinical trials related to mTOR inhibitors will be discussed in the section and summarized in **Table 1**.

**Table 1:** Non-exhaustive summary of the application of mTOR inhibitors in clinical trials.

Disease	Study profile	Patient	Arm	Main result	Decision on mTORI	Ref
RCC	Single-arm, phase II study	Metastatic ccRCC patients with first-line therapy failing	Bevacizumab + TEM	6,12,24-month PFS rates: 50.9%, 19.8%, and 5.7% respectively Median PFS and OS: 6.8 months and 18.2 months respectively	Approval	NCT01264341 (221)
	Prospective phase IIa trial	Advanced non-cc RCC	1) TEM; 2) Sunitinib	PFS was 9.3 vs 13.2 months; tumor control rate was 58% vs 90%	Disapproval	NCT00979966 (222)
	Randomized phase III trial	Advanced RCC	1) Lenvatinib + pembrolizumab; 2) Lenvatinib + EVE; 3) Sunitinib	PFS and adverse event rates in group 1 to 3 were 23.9, 14.7, 9.2 months and 82.4%, 83.1%, 71.8 respectively.	Disapproval	NCT02811861 (223)
	Randomized, open-label, phase III trial	Advanced ccRCC	1) Nivolumab; 2) EVE	OS and ORR were 25.8 vs 19.7 months and 23% vs 4% and PFS favored nivolumab (HR, 0.84; 95% CI, 0.72-0.99)	Disapproval	NCT01668784 (224)
	Randomized, double-blind, phase III trial	RCC with a full surgical resection;	1) EVE; 2) placebo	RFS: 67% vs 63% (HR 0.85; 95% CI, 0.72-1.00, p = 0.051)	Disapproval	NCT01120249 (225)
Breast cancer	Randomized trial	Postmenopausal women with ER-positive, HER2-negative breast cancer	1) EVE + letrozole; 2) Fluorouracil + epirubicin + cyclophosphamide	Ultrasound response rate was 65.0% vs 40.0% and relevant biomarkers favored neoadjuvant Eve plus letrozole (Treg/CD4^+^ T cell, PD-L1; Ki67 index; tumor-infiltrating Tregs)	Approval	NCT02742051 (226)
	Open-label phase II randomized clinical trial	Premenopausal women with HR-positive/ErbB2-negative breast cancer	1) EVE + letrozole; 2) letrozole	Median PFS: 19.4 vs 12.9 months (HR 0.64; 95% CI, 0.46-0.89)	Approval	NCT02313051 (227)
	Randomized phase II trial	Advanced HER2-negative breast cancer	1) Vinorelbine + EVE; 2) Vinorelbine	Median PFS and OS were 4.01 vs 4.08 and 16.3 vs 13.8 months and the PFS rate at 6 months was 39.4% vs 36.6%.	Disapproval	NCT01520103 (228)
	Randomized double-blind phase III study	Women with high-risk, HR-positive, EGFR 2-negative breast cancer	1) EVE; 2) Placebo All patients received endocrine therapy	DFS at 3 years were 88% and 89% but the adverse event rate was 29.9% and 15.9% in arms 1 and 2 respectively.	Disapproval	NCT01805271 (229)
Bladder cancer	Randomized double-blind trial	High-grade non-muscle invasive bladder cancer	1) Placebo; 2) Rapamycin 0.5mg; 3) Rapamycin 2.0mg;	Median percentage change in BCG-specific γδ T cells was -26%, 9.6%, and 78.85 in arms 1 to 3 respectively, and a significant increase in IL-8 and TNF-α was found on rapamycin 2.0mg	Approval	NCT02753309 (230)
	Phase I/II trial	Muscle-invasive bladder cancer	Rapamycin	Rapamycin is well-tolerated and safe but the complete response rate (23%) failed to meet the objective level (26%)	Disapproval	(231)
Neuro-endocrine tumor	Open-label, phase II study	Advanced, recurrent or metastatic pancreatic neuroendocrine tumor	TEM + bevacizumab	Median PFS was 7.1 months and 6-month PFS was 48%; 54% of patients discontinued due to adverse events	Disapproval	NCT01010126 (232)
Glioma	Phase II study	Children with progressive low-grade glioma	EVE	2- and 3-year PFS were 39 ± 11% and 26 ± 11%. OS was 93 ± 6%.	Approval	NCT00782626 (233)
HNSCC	Randomized, phase II trial	Advanced-stage HNSCC	1) EVE; 2) placebo	PFS favored Eve with no statistical significance; PFS of p16-negative and TP53-mutated patients increased significantly in Eve	Partial support	NCT01111058 (234)
Endometrial cancer	Open-label, phase 1/2 randomized trial	HR-positive recurrent or metastatic endometrial cancer	1) Vistusertib + anastrozole; 2) anastrozole	PFS rate at 8 weeks was 67.3% vs 39.1%; Median PFS was 5.2 vs 1.9 months; ORR was 24.5% vs 17.4%	Approval	NCT02730923 (235)
Ovarian carcinoma	Phase 2, randomized trial	Resistant or refractory ovarian high-grade serous carcinoma	1) paclitaxel + Vistusertib; 2) placebo	No difference in PFS, OR, or response rate	Disapproval	ISRCTN 16426935 (236)
Kidney transplant	Prospective, open-label trial	*De novo* renal transplant recipients	1) EVE + low-dose CNI; 2) mycophenolate + standard-dose CNI	The regime of EVE plus low-dose CNI achieved noninferiority in tBPAR or eGFR <50 mL/min per 1.73 m (2) and lowered the incidence of donor-specific antibodies and viral infections	Approval	NCT01950819 (237)
	Randomized controlled trial	Recipients after 4-month post-transplant therapy	1) Tacrolimus; 2) EVE;	The graft survival rate was 87.50% and 92.86% respectively.	Approval	(238)
	Randomized controlled trial	Adult kidney transplant recipients	1) EVE + tacrolimus; 2) Tacrolimus	Fibrosis scores, acute rejection rate, and graft function between the two arms were similar. Eve reduced CMV and BK infection.	Partial support	NCT02096107 (239)
CMV infection	Randomized, open-label phase 4 trial	CMV seropositive kidney transplant recipients	1) EVE; 2) MPA;	Fewer patients required CMV treatment in the Eve group (21.8% vs 47.1%, p = .0007)	Approval	NCT02328963 (240)
GVHD	Phase 2 trial	Patients with hematologic malignancies treatable by allogeneic HCT	CsA, MMF, and SRL	Cumulative incidence of GVHD at day 100 was 36% meeting the primary end point and overall survival at 4 years was 36%.	Approval	NCT01251575 (241)
	Randomized, phase 3 trial	Advanced hematological malignancies treatable by allogeneic HSCT	1) SRL + CsA + MMF; 2) CsA + MMF	Cumulative incidence of GVHD at day 100 was 26% vs 52%; non-relapse mortality was significantly lower in the sirolimus group; OS and PFS were significantly higher in the sirolimus group	Approval	NCT01231412 (242)
Heart Transplant	Prospective, randomized, open-label trial	*De novo* adult heart transplant recipients	1) EVE + CsA + MMF + corticosteroids; 2) CsA + MMF + corticosteroids	After 36 months, systolic blood pressure decreased in the EVE group significantly (p = 0.02)	Approval	NCT01266148 (243)
IPF	Randomized double-blind trial	Patients with IPF	1) SRL; 2) placebo	Sirolimus reduced total fibrocytes, CXCR4+ fibrocytes, and fibrocytes expressing α-smooth muscle actin significantly with fewer side effects.	Approval	NCT01462006 (244)
Pulmonary tuberculosis	Prospective, open-label, phase 2, randomized trial	Patients with pulmonary tuberculosis	1) CC-11050; 2) EVE; 3) Auranofin; 4) Ergocalciferol	The treatment of CC-11050 or everolimus restored FEV1 significantly with no adverse events reported.	Approval	NCT02968927 (245)
ALS	Randomized, double-blind trial	ALS patients	1) Rapamycin 2mg; 2) Rapamycin 1 mg; 3) Placebo	Rapamycin downregulated the expression of IL-18 and the percentage of monocytes and memory-switched B cells	Approval	NCT03359538 (246)
MSA	Randomized, double-blind trial	Patients with probable MSA	1) SRL; 2) placebo	No difference in UMSARS score, neuroimaging, and biomarker was observed; more adverse events occurred in the sirolimus group.	Disapproval	NCT03589976 (247)
Depression	Phase I/II trial	Patients suffering a major depressive episode	1) Rapamycin + ketamine; 2) Placebo + ketamine;	Response and remission rates following rapamycin + ketamine were higher compared to placebo + ketamine (41% vs 13%, p = 0.04, and 29% vs 7%, p = 0.003, respectively)	Approval	NCT02487485 (248)
ITP	Randomized blinded trial	Pediatric patients over 5 years old with chronic ITP	1) SRL; 2) CsA	Both agents showed similar effectiveness in increasing the number of platelets. Sirolimus was safer than CsA	Approval	IRCT20180501039499N1 (249)
CTD-TP	Single-arm, phase II study	Refractory CTD-TP patients	SRL administration	60% of patients achieved a 50% complete remission rate with no adverse events reported	Approval	NCT03688191 (250)
RA	Randomized, controlled trial	Patients with RA	1) Conventional treatment + SRL; 2) Conventional treatment	Significant reductions in disease activity indicators and higher levels of Tregs were observed in patients treated with sirolimus	Approval	ChiCTR-IPR-17010307 (251)
TSC	Randomized, double-blind trial	Children with TSC and IQ <80, learning disability, special schooling, or autism, aged 4-17 years	1) EVE; 2) placebo	Everolimus did not affect IQ, autism, neuropsychological functioning, and behavioral problems.	Disapproval	NCT01730209 (252)
Slow-flow vascular malformation	Open-label, observational-phase randomized trial	Children aged 6 to 18 years with a slow-flow vascular malformation	Patients underwent an observational period and then received SRL.	Sirolimus failed to change the volume of vascular malformations but improved the symptoms, including pain, bleeding, oozing, self-assessed efficacy, and life quality.	Approval	NCT02509468 (253)

mTORI, mTOR inhibitor; TEM, temsirolimus; SRL, sirolimus; EVE, everolimus; PFS, progression free survival; OS, overall survival; ORR, objective response rate; RCC, renal cell cancer; ccRCC, clear cell renal cell cancer; RSS, recurrence free survival; ER, estrogen receptor; HER2, human epidermal growth factor receptor 2; EGFR2, epidermal growth factor receptor 2; DFS, disease free survival; HNSCC, head and neck squamous cell carcinoma; HR, hormone receptor; tBPAR, treated biopsy-proven acute rejection; eGFR, estimated glomerular filtration rate; CNI, calcineurin inhibitor; MPA, mycophenolic acid; CsA, cyclosporine; MMF, mycophenolate mofetil; CMV, cytomegalovirus; GVHD, graft-versus-host disease; HCT, hematopoietic cell transplantation; HSCT, hematopoietic stem cell transplantation; IPF, idiopathic pulmonary fibrosis; FEV, forced expiratory volume; ALS, amyotrophic lateral sclerosis; MSA, multiple system atrophy; ITP, immune thrombocytopenia; CTD-TP, connective tissue disease-related refractory thrombocytopenia; RA, rheumatoid arthritis; TSC, tuberous sclerosis complex.

### 6.1 mTOR inhibitors in organ transplant

Despite preclinical experiments confirming the role of mTOR inhibitors in a variety of diseases, most clinical trials focus on their application in organ transplant as a potent immunosuppressant. In terms of graft-versus-host disease (GVHD) prophylaxis, the addition of sirolimus to standard therapy is propitious to reducing the incidence and severity of graft-versus-host disease (GVHD) and improving the life quality in kidney transplant recipients (241, 242, 254, 255). Additionally, everolimus improves the prognosis of liver or heart transplants with fewer adverse effects (256, 257). Since the use of immunosuppressant increases the susceptibility to infections, whether the administration of mTOR inhibitors influence infections has been paid attention to in organ transplant. Tedesco-Silva, H. et al. compared the efficacy of everolimus and low-dose calcineurin inhibitor with standard therapy (calcineurin inhibitor and mycophenolic acid) and found that the incidence of cytomegalovirus (CMV) infection decreased significantly in patients treated with everolimus (258). Moreover, sirolimus also prevents CMV recurrence in CMV-positive kidney transplant recipients (259). Hence, mTOR inhibitor is an effective and safe therapeutic strategy in organ transplant.

### 6.2 mTOR inhibitors in antitumor therapy

The value of mTOR inhibitors in antitumor therapy has been investigated, especially in urinary tumors. A phase II clinical trial confirmed that combining cyclophosphamide and everolimus leads to the depletion of Tregs and myeloid-derived suppressor cells, which improves the survival of metastatic renal cell cancer (RCC) patients (260). Another phase 2 study proved that the combination of lapatinib plus everolimus is effective in restricting the expansion of tumors with controllable side effects in non-clear cell RCC (261). However, the effectiveness of mTOR inhibitors in treating RCC remains controversial. A phase 3 trial exhibited a longer recurrence-free survival in patients receiving everolimus than those treated with placebo but the result failed to meet the statistical requirement and support the adjuvant treatment of everolimus for RCC (225). Other regimens display improved therapeutic efficacy over mTOR inhibitors in RCC. Lenvatinib plus pembrolizumab prolonged the progression-free and overall survival and improved health-related quality-of-life in patients with advanced RCC compared with Lenvatinib plus everolimus (223, 262). In addition to RCC, the immunomodulatory effect of mTOR inhibitors has been employed in the treatment of bladder cancer. The count of γδT cells specific to BCG and the production of cytokines are boosted by rapamycin, suggesting that rapamycin is a potential adjuvant agent of bladder cancer by enhancing the immune response to BCG to exert antitumor effect (230).

Moreover, the application of mTOR inhibitors has been investigated extensively in breast cancer and other tumors, but few researchers have reached a supportive or positive conclusion. Triple treatment composed of ribociclib, everolimus, and endocrine therapy shows a clinical benefit with manageable safety profile in advanced breast cancer (263). Everolimus plus letrozole or exemestane benefits the outcome and has lower adverse effects in postmenopausal advanced breast cancer patients with hormone receptor-positive and human epidermal growth factor receptor 2-negative (226, 264). Nonetheless, several clinical trials refute the therapeutic efficacy of everolimus in survival improvement in breast cancer (229, 265, 266). A randomized phase II trial demonstrated that the treatment of everolimus and letrozole prolonged the progression-free survival in recurrent endometrial carcinoma (267). Inhibiting PI3K/AKT by everolimus or temsirolimus is tolerable and beneficial to overall survival in glioma and glioblastoma patients (233, 268).

### 6.3 mTOR inhibitors in other disorders

mTOR inhibitor is a promising therapeutic target for autoimmune diseases. Sirolimus downregulates the indicators of disease activity and elevates Tregs in patients with rheumatoid arthritis more significantly than conventional therapy (251). Over half of patients with connective tissue disease-related thrombocytopenia (CTD-TP) who received oral sirolimus administration achieved complete remission, suggesting that sirolimus is an alternative in treating CTD-TP (250). Similarly, sirolimus increases the count of platelets in pediatric chronic immune thrombocytopenia (249).

Furthermore, mTOR inhibitors can also be used as adjuvant agents to augment the efficacy of conventional drugs in treating tuberculosis. Researchers evaluated the lung function and sputum culture to compare the efficacy of different adjuvants plus standard tuberculosis treatment, concluding that everolimus is a potential adjuvant in tuberculosis therapy with safety and well-tolerance (245). In terms of respiratory diseases, the treatment of sirolimus contributes to a significant decrease in circulating fibrocytes to alleviate lung fibrosis, which implies the effect of sirolimus on treating idiopathic pulmonary fibrosis (244).

Regarding neurological disorders, mTOR is a promising therapeutic target as well. Patients of ALS receiving rapamycin underwent a decline in proinflammatory cytokines and an increase in Tregs to inhibit aberrant neuroinflammation (246).

## 7 Conclusion

The precise regulation of cell death is principal to sustaining equilibrium, and abnormal cell death is closely associated with various disorders. As a key molecule in cellular metabolism, growth, and death, mTOR plays an essential role in regulating the complex signaling network. There have been increasing studies that have demonstrated the vital effect of necroptosis regulated by mTOR on a wide range of diseases, such as IBD, sepsis, lung cancer, and others.

We summarized the upstream and downstream mechanisms of mTOR and the molecular mechanisms of necroptosis (**Graphical Abstract**). Recent advances in the mechanisms by which mTOR regulates necroptosis are also highlighted in this review. mTOR, established as a presentative autophagy inhibitor, controls the interaction of RIPKs and their removal through autophagy to regulate necroptosis in a roundabout manner. Several necroptosis-relevant molecules are directly regulated by mTOR, such as RIPKs and TNFα. mTOR also utilizes oxidative stress to control necroptosis. However, subsequent investigations have suggested that mTOR exerts a bidirectional effect on necroptosis. It is determined by numerous factors, such as the stimulus category and cell types. Distinct signaling pathways in which mTOR is involved in the regulation of necroptosis are likely to lead to diverse outcomes; the ultimate effect is contingent upon the dominant mechanism.

Investigations into the mechanisms by which mTOR regulates necroptosis have shed more light on potential therapeutic targets for an extensive array of diseases, including immune-related diseases, cancer, drug toxicity, and more. An incremental body of basic research substantiates the value of targeting mTOR in treating various disorders. Based on the fundamental findings, considerable clinical trials have attempted to apply mTOR inhibitors in therapeutic strategies for transplant rejection, tumor, autoimmune diseases, etc. and presented promising prospects.

Therefore, as a pivotal hub for regulating cell activities, mTOR exerts an essential role in controlling necroptosis through autophagy and other mechanisms. Necroptosis is under the bidirectional regulation of mTOR by virtue of complicated cellular signaling networks but the underlying mechanisms require further investigation. Although both basic experiments and clinical trials have indicated the therapeutic implications of mTOR mediated by necroptosis, substantial evidence from mechanism studies and clinical trials is in enormous demand to demonstrate its therapeutic value and ultimately fulfil clinical transformation of mTOR in various diseases such as cancer, sepsis, and immune-related disorders.

## Author contributions

YX: Visualization, Writing – original draft, Writing – review & editing. GZ: Writing – review & editing. XL: Writing – review & editing. NC: Conceptualization, Supervision, Writing – review & editing. HW: Conceptualization, Writing – review & editing.
